# Identification of mRNA-, circRNA- and lncRNA- Associated ceRNA Networks and Potential Biomarkers for Preeclampsia From Umbilical Vein Endothelial Cells

**DOI:** 10.3389/fmolb.2021.652250

**Published:** 2021-04-20

**Authors:** Dan Chen, Biwei He, Panchan Zheng, Shuying Wang, Xueya Zhao, Jinyu Liu, Xingyu Yang, Weiwei Cheng

**Affiliations:** ^1^International Peace Maternity and Child Health Hospital, School of Medicine, Shanghai Jiao Tong University, Shanghai, China; ^2^Shanghai Key Laboratory of Embryo Original Disease, Shanghai, China

**Keywords:** preeclampsia, HUVECs, circRNAs, ceRNA network, biomarker

## Abstract

**Objective:**

The etiology and pathogenesis of preeclampsia (PE) remain unclear, and ideal biomarkers for the early detection of PE are scarce. The involvement of the competing endogenous RNA (ceRNA) hypothesis in PE is only partially understood. The present study aimed to delineate a regulatory network in PE comprised of messenger RNAs (mRNAs), circular RNAs (circRNAs), long non-coding RNAs (lncRNAs), and microRNAs (miRNAs) via ceRNA profiles from human umbilical vein endothelial cells (HUVECs) to further reveal the pathogenesis of PE and potential biomarkers.

**Methods:**

Differentially expressed mRNAs, circRNAs, and lncRNAs were detected in HUVECs from early onset preeclampsia (EOPE) cases (*n* = 4) and normal pregnancies (*n* = 4) by microarray analysis. Bioinformatics analysis was performed to systematically analyze the data, and a relevant ceRNA network was constructed. RNAs (*ANGPT2*, *LIPG*, *hsa_circ_0025992*, *hsa_circ_0090396*, *hsa_circ_0066955*, *hsa_circ_0041203*, *hsa_circ_0018116*, *lnc-C17orf64-1:1*, *lnc-SLC27A2-2:1*, and *lnc-UEVLD-5:1*) were validated by quantitative real-time PCR (qRT-PCR) in 10 pairs of HUVECs and placental tissues from PE patients and normal pregnancies. Furthermore, expression of *hsa_circ_0025992* was detected in maternal peripheral blood samples from PE patients (*n* = 24) and normal pregnancies (*n* = 30) to confirm its potential as a novel biomarker. The receiver operating characteristic (ROC) curve was applied to analyze its diagnostic value.

**Results:**

Compared with HUVECs from normal pregnancies, HUVECs from EOPE cases had 33 differentially expressed mRNAs (DEmRNAs), 272 DEcircRNAs, and 207 DElncRNAs. GO and KEGG analyses of the DERNAs revealed the biological processes and pathways involved in PE. Based on the microarray data and the predicted miRNAs, a ceRNA network was constructed with four mRNAs, 34 circRNAs, nine lncRNAs, and 99 miRNAs. GO and KEGG analyses of the network reinforced the crucial roles of metabolic disorders, the p53 and JAK/STAT signaling pathways in PE. In addition, ROC analysis indicated that *hsa_circ_0025992* could be used as a novel biomarker for PE.

**Conclusion:**

A novel ceRNA network was revealed in PE, and the potential of *hsa_circ_0025992* to serve as a new biomarker was confirmed.

## Introduction

Preeclampsia (PE), a systemic vascular disorder specific to pregnancy, is traditionally characterized by hypertension and proteinuria after 20 weeks of gestation (ACOG, 2020). PE affects approximately 3–5% of pregnancies, leading to maternal and fetal morbidity and mortality (Mol et al., 2016). The etiology of PE remains largely unknown; however, it is widely acknowledged that inadequate trophoblast invasion, spiral uterine artery remodeling, endothelial dysfunction, and inappropriate angiogenesis could participate in PE (Ives et al., 2020). Due to the lack of effective treatments, it is essential to identify biomarkers for early diagnosis and therapeutic targets for the precise treatment of preeclampsia (Maric-Bilkan et al., 2019). Endothelial dysfunction is a crucial pathological feature of preeclampsia, which could lead to proteinuria and coagulation dysfunction in patients (Ives et al., 2020). In addition, preeclampsia is regarded as a risk factor for long-term postpartum cardiovascular disease (CVD) (Benschop and Duvekot, 2019; Lane-Cordova et al., 2019). Growing evidence suggests that vascular endothelial dysfunction is critical for the pathogenesis of preeclampsia (Cubro et al., 2020; Mauro et al., 2020; Vicen et al., 2020). Immunological changes (Aneman et al., 2020), metabolic syndrome (Scioscia et al., 2015; Vicen et al., 2020), and endothelial dysfunction are intertwined in preeclampsia. Nevertheless, the exact mechanism of endothelial dysfunction in preeclampsia still needs to be fully elucidated.

The competing endogenous RNA (ceRNA) hypothesis surmises an interaction network between transcripts that share the same microRNA response elements (MREs) (Salmena et al., 2011). Long non-coding RNAs (lncRNAs) are transcripts longer than 200 nt that can regulate neighboring and overlapping coding gene expression in various manners, such as *cis-* and *trans-*regulation (Chen, 2016). Circular RNAs (circRNAs) are novel single-stranded and covalently closed molecules that negatively affect miRNAs by acting as miRNA sponges (Li et al., 2018). The functions of lncRNAs and circRNAs might be embodied in the corresponding mRNA genes and host genes. Additionally, lncRNAs and circRNAs can function as ceRNAs and regulate gene expression (Chen, 2016; Li et al., 2018).

Increasing evidence has revealed that lncRNAs and circRNAs are significantly involved in the pathogenesis of PE through the placental ceRNA networks. *Linc00511* was reported to exert its inhibitory effect on trophoblast invasion through the *miR-29b-3p*/*Cyr61* axis (Quan et al., 2019). *LncRNA AK002210* was found to participate in the progression of PE by affecting the *miR-590-3p/NAIP* and ERK/MMP signaling pathways (Zhao L. et al., 2020). The higher expression of *lncRNA PSG10P* contributed to PE pathogenesis by regulating *miR-19a-3p* and *IL1RAP* (Wang et al., 2019). *CircTRNC18* and *circZDHHC20* sponge *miR-762* and *miR-144*, respectively, affect the expression of *GRHL2* and repress migration of trophoblast cells (Shen et al., 2019; Zhou et al., 2020). Due to their unique circular structure, circRNAs are resistant to ribonucleases and are more stable in body fluids or exosomes than linear RNAs. Moreover, circRNAs are abundant and specifically expressed in tissues and developmental stages (Li et al., 2018). Herein, circRNAs exhibit ideal potential for use as biomarkers for diagnosis. *Hsa_circ_0036877* was identified as a ceRNA and proved to be a potential blood biomarker for PE after receiver operating characteristic (ROC) curve analysis (Hu et al., 2018). To our knowledge, differentially expressed non-coding RNAs were often identified from placental tissues, maternal blood plasma samples, and umbilical cord blood plasma samples, and could affect the angiogenesis ability of the HUVECs *in vitro* (Xu et al., 2017; Lip et al., 2020; Zhao X. et al., 2020). A study revealed HUVEC-originated miRNAs in PE and suggested that the PE-downregulated miR-29a/c-3p may inhibit endothelial cell migration through the FGF2-activated PI3K-AKT signaling pathway (Zhou et al., 2017). However, there was no research on HUVEC-originated ceRNA networks in PE.

To offer evidence of the relationship between endothelial dysfunction and preeclampsia, our study applied ceRNA microarray analysis in primary HUVECs extracted from PE patients and normal pregnancies. Then, we tried to illuminate the lncRNA/circRNA–miRNA–mRNA network with bioinformatics analysis. Furthermore, a genetic biomarker of PE in the blood needs to be identified.

## Materials and Methods

### Study Design and Sample Collection

This research was divided into two parts. First, the microarray assay was conducted with four pairs of HUVECs extracted from patients with EOPE and normal pregnancies, and 10 pairs of HUVECs and placental tissues were collected for validation. Second, a preliminary delineation of the ceRNA profiling and a prospective study on *hsa_circ_0025992* in maternal whole peripheral blood from PE patients (*n* = 24) and normal pregnant women (*n* = 30) were performed to predict PE at 11–12 weeks of gestation. HUVECs and placental tissues were collected from participants who had cesarean section. Maternal whole peripheral blood samples were collected in the first trimester and selected after the diagnosis of PE. Placental tissues and blood samples were stored at −80°C until use. All samples were collected with participants’ informed consent at the International Peace Maternity and Child Health Hospital, Shanghai, China. The research was authorized and approved by the hospital ethics committee. The diagnosis of preeclampsia was based on the guidelines from the American College of Obstetricians and Gynecologists (ACOG) (ACOG, 2020), and participants with multiple gestations, chronic hypertension, gestational diabetes mellitus, thyroid dysfunctions, and kidney diseases were eliminated. The clinical characteristics are described in Tables 1, 2.

**TABLE 1:** Clinical characteristics of EOPE and controls for microarray.

	**Age (year)**	**BMI (kg/m^2^)**	**Week gestation (week)**	**Systolic BP (mmHg)**	**Diastolic BP (mmHg)**	**Proteinuria (g/24 h)**	**Birth weight (g)**
C1	29	21.1	38.71	105	60	–	3685
C2	24	20.9	38.57	120	80	–	3495
C3	29	16.8	38.71	118	73	–	3230
C4	30	21.9	39.14	120	74	–	3080
EOPE1	29	22.8	34.29	141	95	0.51	2200
EOPE2	40	21.9	28.57	171	111	2.61	1120
EOPE3	38	22.0	31.00	171	100	2.40	1425
EOPE4	27	18.8	34.00	155	90	1.53	1880
*P*-value	0.1908	0.4382	0.0147	0.0286^#^	0.0044	0.0286^#^	0.0008

*C, control; EOPE, early onset preeclampsia; BMI, body mass index; BP, blood pressure. #: the Mann–Whitney *U* test.*

**TABLE 2:** Clinical characteristics of samples for validation.

**Characteristics**	**CON (*n* = 10)**	**PE (*n* = 10)**	***P*-value**
Age, year	30.40 ± 4.30	33.10 ± 5.82	0.2534
BMI	21.42 ± 3.31	22.62 ± 3.15	0.4175
Gestation, week	38.83 ± 0.43	34.34 ± 3.24	0.0017
Systolic blood pressure, mmHg	116.40 ± 7.21	156.30 ± 13.61	<0.0001
Diastolic blood pressure, mmHg	71.10 ± 6.72	95.40 ± 8.61	<0.0001
Proteinuria, g/24 h	–	1.43 ± 0.96	0.0011
Birth weight, g	3314.50 ± 370.39	2069.50 ± 582.01	<0.0001

*Data were shown as mean ± SD.*

### Primary HUVEC Isolation and Identification

Primary HUVECs were isolated from umbilical cords within 2 h after cesarean section under aseptic conditions. After washing the umbilical vein with PBS (HyClone, United States), 30 ml of 0.1% type I collagenase solution (Gibco, United States) was injected into the umbilical vein. Both extremities of the vein were clamped, and the cord was incubated at 37°C and 5% CO_2_ for 15 min. Subsequently, the cord was squeezed, the contents were transferred to a sterile 50 ml tube, and the complete endothelial cell growth medium (ECM; ScienCell, United States) was added. The tubes were centrifuged at 1000 rpm for 10 min. Finally, the cells were suspended and transferred to a culture dish precoated with 2% gelatin (Sigma-Aldrich, United States). When 50–60% confluence was achieved, the cells were identified by an immunofluorescence assay with anti-CD31 (CST, United States), anti-factor VIII (Bioss, United States), and DAPI (Yeasen, China) staining. CD31 and factor VIII expression was positive in HUVECs. The identification results were shown in Supplementary Figure 1.

### RNA Purification and Labeling

Total RNA from primary HUVECs was extracted and purified utilizing mirVana^TM^ miRNA Isolation Kit (Ambion, United States) according to the manufacturer’s instructions. The concentration of RNA was detected by a NanoDrop ND-2000 spectrophotometer (Thermo Fisher Scientific, United States), and RNA integrity was evaluated by an Agilent Bioanalyzer 2100 (Agilent, United States) with the RNA integrity numbers (RINs) ≥7.0 and 28 s/18 s ≥0.7. Qualified total RNA was further purified by a RNeasy Mini Kit (Qiagen, Germany) and RNase-Free DNase Set (Qiagen, Germany). Total RNA was amplified and labeled using a Low Input Quick Amp WT Labeling Kit (Agilent, United States). In addition, a RNeasy Mini Kit was used to purify the labeled cRNAs.

### Microarray Assay and Data Acquisition

The SBC Human (4 × 180K) ceRNA array V1.0 (SHBIO, China) assay was used to detect differentially expressed mRNA, circRNA, and lncRNA profiles in HUVECs by comparing normal pregnancies with PE patients (*n* = 4). According to the manufacturer’s instructions, each slide was hybridized with 1.65 μg of Cy3-labeled cRNA for 17 h using a Gene Expression Hybridization Kit (Agilent, United States) in a hybridization oven (Agilent, United States). Then, the slides were washed in staining dishes (Thermo Fisher Scientific, United States) by Gene Expression Wash Buffer Kit (Agilent, United States) and scanned exploiting an Agilent Microarray Scanner (Agilent, United States) with the default settings. Raw data were obtained by Feature Extraction software 12.0 (Agilent, United States) and normalized with the quantile algorithm of the limma package in R^1^. The data were analyzed with Student’s *t*-test, | fold change| (| FC|) ≥ 2 and *P*-value < 0.05 were set as the cutoff criteria.

### Bioinformatics Analysis

#### Hierarchical Clustering Analysis

Hierarchical clustering was conducted by the Cluster and Tree View program in R^1^ software to identify and visualize the overview of the DERNA profiles based on the microarray dataset.

#### LncRNA/circRNA Targeting Genes and MREs Prediction

Long non-coding RNAs and their potential target genes were visualized based on UCSC^2^. *Cis-*regulated genes were defined as protein-coding genes located on the same chromosome within a 10 kb region upstream or downstream of the corresponding lncRNAs. *Trans-*regulated genes were selected by two steps: (1) Complementary or similar sequences were first selected by BLAST^3^ from the same species database; (2) The complementary energy between the two sequences was calculated by using RNAplex, and the genes with *E* ≤ −30 were selected as the *trans-*regulated genes of the lncRNAs. The host genes of the circRNAs obtained from the CircBase^4^ were regarded as their target genes. And the putative MREs of differentially expressed lncRNAs and circRNAs were identified by bioinformatics analysis with Miranda^5^.

#### GO and KEGG Pathway Analysis

Gene ontology (GO) and Kyoto Encyclopedia of Genes and Genomes (KEGG) pathway analyses were performed with Fisher’s exact-test by the clusterProfiler package in R^1^ software. The standard of selection was the number of genes that fell on a GO term or pathway ≥2, with *P*-value < 0.05. Significantly enriched GO terms and KEGG pathways are presented.

#### CeRNA Network Construction

Based on the ceRNA hypothesis, combined with the microarray data (DElncRNAs, DEcircRNAs, DEmRNAs, and lncRNA/circRNA targeting genes) and the intersecting predicted miRNAs, the construction of the ceRNA network was visualized and analyzed via Cytoscape_V2_8_3.

### Quantitative Real-Time PCR Analysis

RNA (800 ng) was reverse transcribed by cDNA synthesis kit (TRANS, China) following the protocol. Specific gene primers in human (Table 3) were designed by NCBI Primer-BLAST^3^. The subsequent qRT-PCR (10 μl volume) was accomplished by a QuantStudio 7 Flex instrument (Thermo Fisher Scientific, United States) with a SYBR Premix Ex Taq Kit (Takara, Japan). Relative RNA expression was calculated by the 2^–ΔΔ*Ct*^ method and normalized to *18S rRNA* expression.

**TABLE 3:** Primer sequences for PCR.

**RNA name**	**RNA type**	**Forward sequence (5′-3′)**	**Reverse sequence (5′-3′)**
*ANGPT2*	mRNA	TCAGTGGCTAATGAAGCTTGAGA	CCGCTGTTTGGTTCAACAGG
*LIPG*	mRNA	GGGAGCCCCGTACCTTTTG	CCTCACAGATGGTTTGACCTCA
*lnc-C17orf64-1:1*	lncRNA	GGCCTGGTCTGGTACTCTGTGAC	TTGGCATGGATAGCAGAGTGGTTG
*lnc-SLC27A2-2:1*	lncRNA	CCCATGGAGCCGGAGATAG	CCAGGGATTTTATGCTGTGGC
*lnc-UEVLD-5:1*	lncRNA	AGTCACGAAGGCAGTCACAG	AGAAGACATGCCAGGAAAGCA
*hsa_circ_0025992*	circRNA	CTGCTTGCCGCCCTCTTTGG	TCACAGCCATTAACACAGCCAGAC
*hsa_circ_0090396*	circRNA	GCCTCTTCAAGCAGCCAGCAG	ACAGCCTCCAGCACCTCCAAG
*hsa_circ_0066955*	circRNA	GAAGAGCCAGTGTACGAAGCAGAG	CTCATGCCTGTCCATCTCCTCAAC
hsa_circ_0041203	circRNA	GGAATGTAGCCGAGAGGTTGTGTG	TGCCAAGGGAATCAGGAGGAGAG
hsa_circ_0018116	circRNA	CATCCAGCAGCTTCTCCAACTCAC	CACTTGTTTGCCTCCTCCACCTC
18S rRNA	mRNA	CAGCCACCCGAGATTGAGCA	TAGTAGCGACGGGCGGTGTG

### Statistical Analysis

Continuous data were presented as means ± standard deviations (SD) unless specifically otherwise indicated. Statistically significant differences were estimated by Student’s *t*-test or the Mann–Whitney *U* test. The receiver operating characteristic (ROC) curve analysis and Spearman correlation analysis were used to evaluate the predictive value of the *hsa_circ_0025992* and the correlation of the circRNA with blood pressure of the patients. All statistical analyses were established by GraphPad Prism 7.0 and SPSS version 20.0. *P*-value < 0.05 was considered as statistically significant.

## Results

### Identification of DERNAs

**FIGURE 1:**
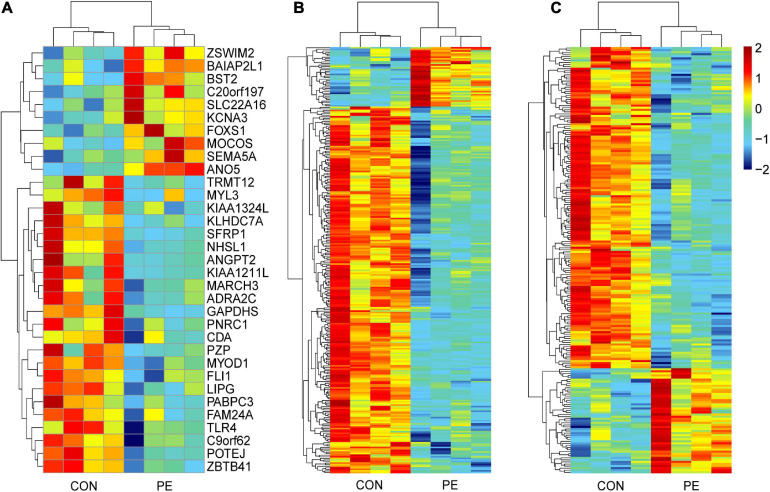
Hierarchical clustering analysis of DERNAs in HUVECs between normal pregnancies and PE patients. The heatmap reveals 33 DEmRNAs **(A)**, 272 DEcircRNAs **(B)**, and 207 DElncRNAs **(C)**. Each column and row correspond to a sample and a transcript, respectively. Red represents upregulation, while blue represents downregulation. CON, control; PE, preeclampsia, *n* = 4.

**FIGURE 2:**
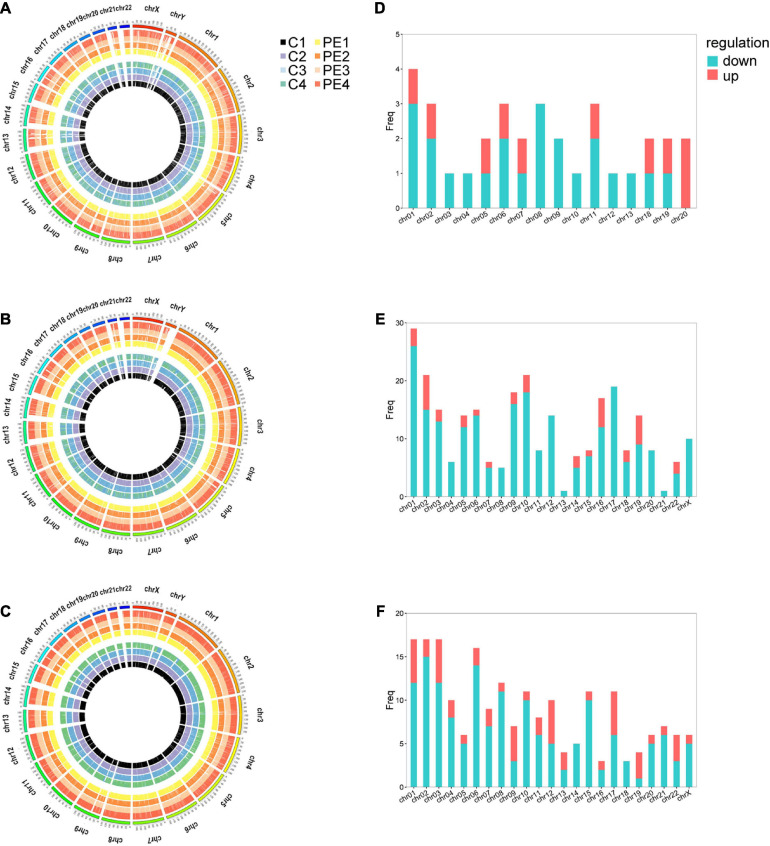
The distribution of DERNAs on chromosomes. Circos plot of genome-wide mRNAs **(A)**, circRNAs **(B)**, and lncRNAs **(C)**. Histograms depict the distribution of DEmRNAs **(D)**, DEcircRNAs **(E)**, and DElncRNAs **(F)** mapped on each chromosome. The outermost circle represents the human chromosomes, while the inside eight circles represent eight samples from control and PE patients. The *Y*-axis denotes the count of transcripts mapped to the chromosome. CON, control; PE, preeclampsia, *n* = 4.

Microarray analysis was performed to explore the pathogenesis of preeclampsia from the perspective of endothelial dysfunction. A total DERNA profile was described after stringent filtering with the following standards: absolute fold change ≥2.0 and *P*-value < 0.05. The analysis demonstrated that 33 DEmRNAs (10 upregulated and 23 downregulated mRNAs, Figure 1A), 272 DEcircRNAs (38 upregulated and 234 downregulated circRNAs, Figure 1B), and 207 DElncRNAs (51 upregulated and 156 downregulated lncRNAs, Figure 1C) were identified. The total microarray-detected RNAs were widely distributed on all human chromosomes, and most of the significantly dysregulated RNAs were downregulated (Figure 2).

The variability between PE samples and controls in the microarray was shown in the volcano plot (Supplementary Figure 2). These aforementioned results illustrated that RNA expression in the PE group was different from that in the control group.

### GO Enrichment Analysis of DERNAs

**FIGURE 3:**
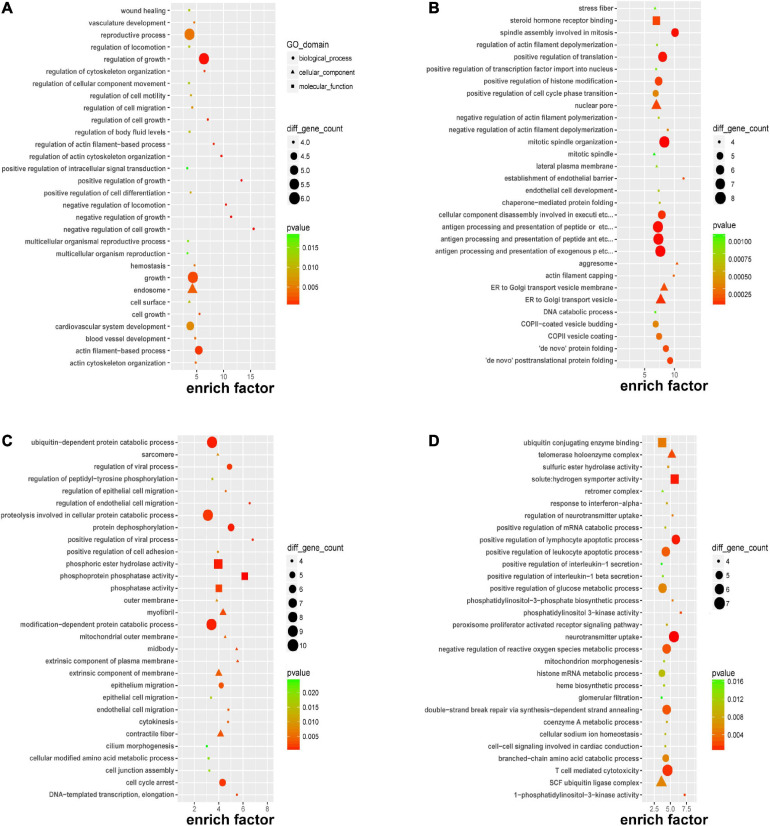
Gene Ontology (GO) enrichment of DERNAs. GO enrichment of DEmRNAs **(A)**, host genes of DEcircRNAs **(B)**, *cis-*regulated genes of DElncRNAs **(C)**, and *trans-*regulated genes of DElncRNAs **(D)**.

To gain further insight into the pathogenesis that was potentially mediated by these DERNAs in PE, GO analysis was performed. Focusing on the biological process, it was revealed that the DEmRNAs mainly affect the regulation of growth and cellular processes through “negative regulation of cell growth,” “positive/negative regulation of growth,” and “negative regulation of locomotion” in Figure 3A; it was suggested that circRNAs might participate in the “establishment of the endothelial barrier,” “*de novo* posttranslational protein folding,” “antigen processing and presentation,” and “mitotic process” in Figure 3B; the *cis-*regulated genes of lncRNAs played a pivotal role in “endothelial and epithelial cell migration” in Figure 3C, and the *trans-*regulated genes of lncRNAs could alert “lymphocyte apoptotic process,” “neurotransmitter uptake,” and “T cell-mediated cytotoxicity” in Figure 3D. Most of the aforementioned biological processes indicated that immune injury, intercellular signaling, and endothelial dysfunction were associated with PE.

### KEGG Pathway Analysis of DERNAs

**FIGURE 4:**
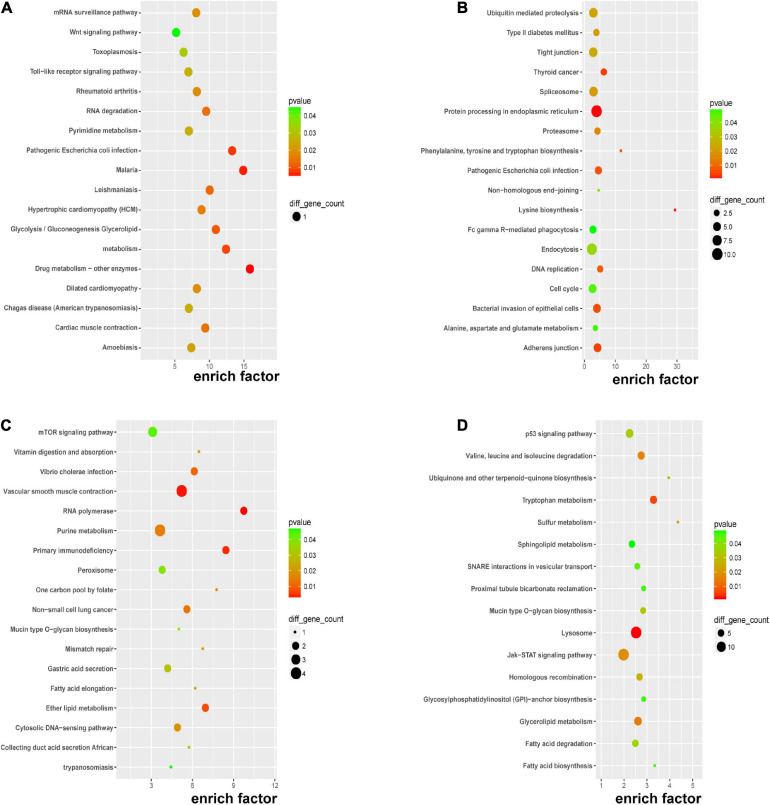
KEGG pathway analysis of DERNAs. KEGG pathway analyses of DEmRNAs **(A)**, host genes of DEcircRNAs **(B)**, *cis-*regulated genes of DElncRNAs **(C)**, and *trans-*regulated genes of DElncRNAs **(D)**.

Kyoto encyclopedia of genes and genomes pathway enrichment analysis for significant DERNAs was executed to comprehend pathways and molecular interactions relevant to genes. As shown in Figure 4A, the top three pathways for DEmRNAs were “drug metabolism-other enzymes,” “malaria,” and “pathogenic *Escherichia coli* infection”. In Figure 4B, the top three pathways for the host genes of DEcircRNAs were “lysine biosynthesis,” “phenylalanine, tyrosine, and tryptophan biosynthesis,” and “thyroid cancer”. In Figure 4C, the top three pathways for *cis-*regulated genes of DElncRNAs were “RNA polymerase,” “primary immunodeficiency,” and “one-carbon pool by folate”; and in Figure 4D, the top three pathways for *trans-*regulated genes of DElncRNAs were “sulfur metabolism,” “ubiquinone and other terpenoid-quinone biosynthesis,” and “tryptophan metabolism”. Major nutrient metabolism disorders were also revealed in Figures 4A,C,D. Collectively, insights into the correlation of immunodeficiency, metabolic disorders, and bacterial dysbiosis with endothelial dysfunction in preeclampsia were proposed.

### Validation of mRNAs, circRNAs, and lncRNAs Expression

**FIGURE 5:**
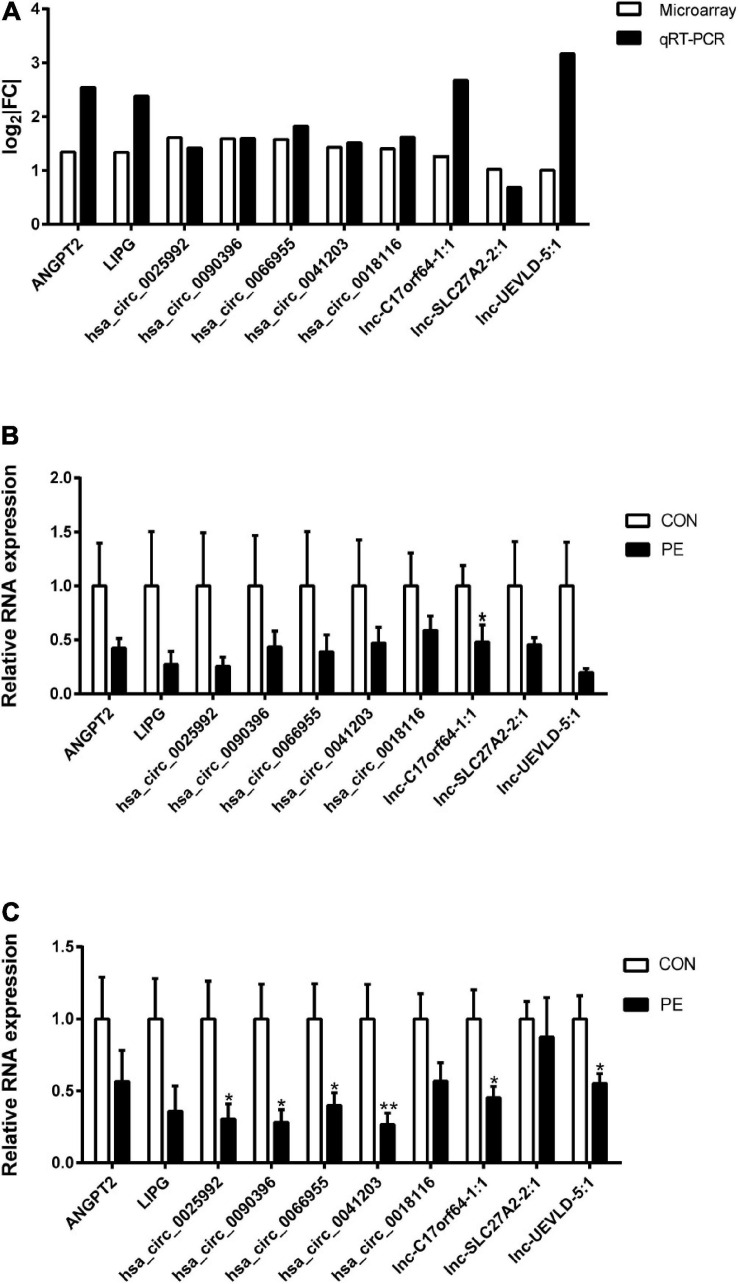
Validation of DERNA expression by qRT-PCR. Differentially expressed mRNAs, circRNAs, and lncRNAs in PE samples by microarray analysis were validated by qRT-PCR **(A)**, *n* = 4. Validation of differentially expressed mRNAs, circRNAs, and lncRNAs in 10 pairs of HUVECs **(B)** and corresponding placental tissues **(C)** of participants. | FC| = | Fold Change|. CON, control; PE, preeclampsia. Data were shown in mean ± SEM. *P < 0.05; **P < 0.01.

To validate the microarray profiling results, two mRNAs (*ANGPT2* and *LIPG*), five circRNAs (*hsa_circ_0025992*, *hsa_circ_0090396*, *hsa_circ_0066955*, *hsa_circ_0041203*, and *hsa_circ_0018116*), and three lncRNAs (*lnc-C17orf64-1:1*, *lnc-SLC27A2-2:1*, and *lnc-UEVLD-5:1*) were selected for verification by quantitative real-time PCR (qRT-PCR) in the same HUVEC samples. The results in Figure 5A showed high consistency between the microarray and qRT-PCR analyses. The expression of the selected DERNAs in HUVECs and corresponding placental tissues from normal participants (*n* = 10) and PE patients (*n* = 10) was determined. The qRT-PCR results in Figures 5B,C showed that the selected genes were decreased in both HUVECs and placental tissues from the PE group; *Lnc-C17orf64-1:1* expression in HUVECs and placenta of the PE group was significantly lower than that in the normal group (Figures 5B,C), and the expression levels of *hsa_circ_0025992*, *hsa_circ_0090396*, *hsa_circ_0066955*, *hsa_circ_0041203*, *lnc-C17orf64-1:1*, and *lnc-UEVLD-5:1* were significantly decreased in placental tissues (Figure 5C). These findings indicated that some DERNAs could be implicated in the pathogenesis of PE.

### Global ceRNA Network Integration in PE

**FIGURE 6:**
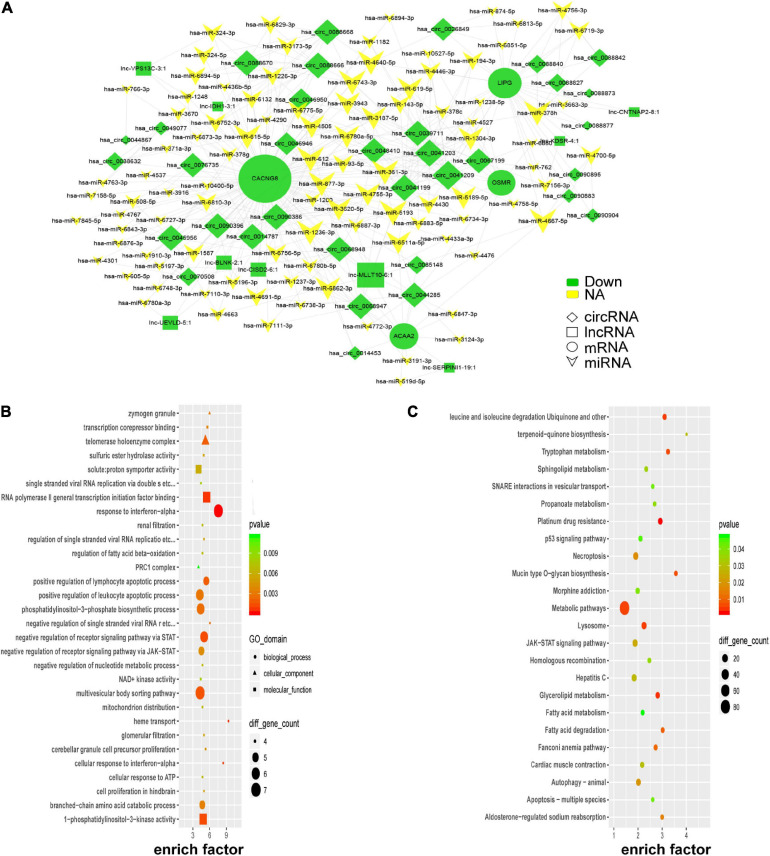
Global ceRNA network integration in PE. A portion of the theoretical ceRNA network in PE was predicted according to the microarray analysis **(A)**. GO analysis of the molecules involved in the ceRNA network **(B)**. KEGG pathway analysis of the molecules involved in the ceRNA network **(C)**.

According to the ceRNA hypothesis, lncRNAs could compete with circRNAs for the same miRNAs and further impact gene expression. Combined with our microarray data and predicted miRNAs, a portion of the ceRNA network was constructed by Cytoscape_V2_8_3 and visualized in Figure 6A. Four key genes (*CACNG8*, *LIPG*, *ACAA2*, and *OSMR*) were identified in the network. To further uncover insights into the ceRNA network, GO, and KEGG enrichment analyses were performed as shown in Figures 6B,C. PE-associated pathways, such as the “p53 signaling pathway,” “JAK-STAT signaling pathway,” “fatty acid degradation,” and “autophagy-animal” were depicted (Gao et al., 2016; Zhao and Jiang, 2018; Nakashima et al., 2020; Khaire et al., 2021).

### *Hsa_circ_0025992* Functions as a Novel Potential Blood Biomarker

**FIGURE 7:**
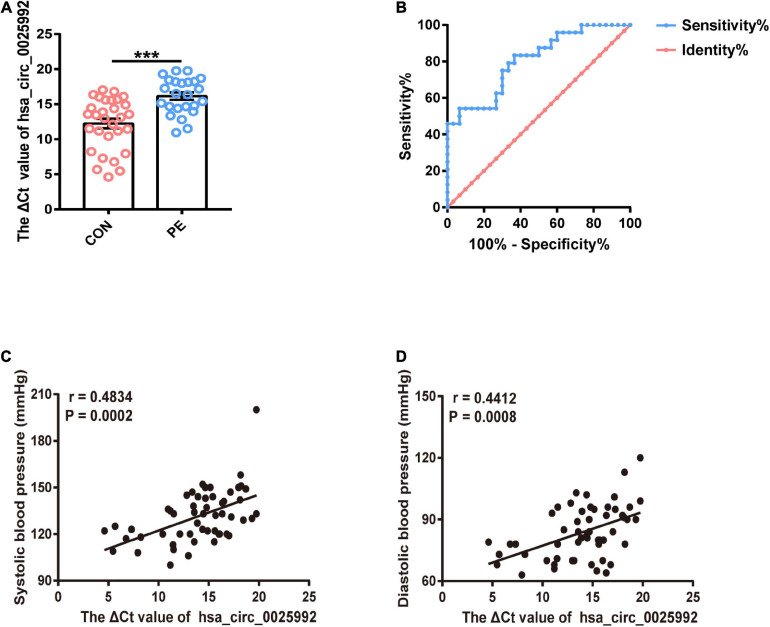
*Hsa_circ_0025992* serving as a potential novel blood biomarker for the prediction of early PE. Expression of *hsa_circ_0025992* in blood samples from PE patients (*n* = 24) and normal pregnant women (*n* = 30) **(A)**. Data were shown as the mean ± SEM. The ROC curve of *hsa_circ_0025992* as a predictive biomarker for early PE. AUC = 0.8065 (95% CI 0.6902–0.9210, *P* = 0.0001); sensitivity and specificity, 54.17 and 93.33%, respectively. The cutoff value was 16.36 (ΔCt value) [the optimal cutoff point was determined at the maximum Youden Index (YI)]. The NPV of a ΔCt value ≤ 16.36 for a diagnosis of preeclampsia was 71.8%. A ΔCt value above 16.36 had a PPV of 86.7% **(B)**. Spearman correlation analysis for *hsa_circ_0025992* (ΔCt value) in blood samples with the systolic blood pressure and diastolic blood pressure of the corresponding participants **(C,D)**. *P < 0.05, **P < 0.01, ***P < 0.001.

According to the microarray results and bioinformatics analysis, *hsa_circ_0025992* showed a significant change, and it was predicted to sponge a reported angiogenic microRNA, *hsa-miR-20a-3p* (Supplementary Table 1). To confirm the potential of *hsa_circ_0025992* as a biomarker, its expression in maternal whole peripheral blood was detected by qRT-PCR in normal pregnancies (*n* = 30) and PE patients (*n* = 24) at 11–12 weeks of gestation. Compared to normal patients, PE patients who were diagnosed based on ACOG at the end of gestation had a significantly higher ΔCt value of *hsa_circ_0025992*, indicating its lower expression level in blood samples (Figure 7A). Furthermore, receiver operating characteristic (ROC) curve analysis was performed, and the area under the ROC curve (AUC) of *hsa_circ_0025992* was 0.8065 [95% confidence interval (CI) 0.6902–0.9210], *P* < 0.001. The cutoff value determined by the maximum Youden Index (YI) was 16.36 (ΔCt value). The sensitivity and specificity were 54.17% and 93.33%, respectively. The measurement of a ΔCt value < 16.36 demonstrated a 71.8% negative predictive value (NPV) for the diagnosis of preeclampsia. A ΔCt value above 16.36 had a relatively high positive predictive value (PPV) of 86.7% (Figure 7B). Spearman correlation analysis depicted that the ΔCt value of *hsa_circ_0025992* positively correlated with the systolic blood pressure and diastolic blood pressure of the corresponding participants (Figures 7C,D). These results revealed that *hsa_circ_0025992* could serve as a novel blood biomarker for the prediction of early PE.

## Discussion

With the rapid development of high-throughput technology and bioinformatics analysis, increasing efforts have been made to uncover the pathology of PE, and the novel ceRNA hypothesis is becoming more noteworthy (Hu et al., 2018; Quan et al., 2019). To the best of our knowledge, there has been scarce research aimed at the ceRNA network in HUVECs to illustrate the mechanism of endothelial dysfunction in PE.

In our study, for the first time, ceRNA profiling of EOPE HUVECs was revealed, and further bioinformatics analysis was plotted to clarify the deregulated biological processes or pathways affected by ceRNAs. A set of DERNAs were identified, validated, and found to be distributed across all chromosomes, denoting that non-coding RNAs are widely implicated in gene regulation. Subsequently, GO enrichment of DERNAs suggested that immune injury substantially linked endothelial dysfunction to PE and thus might affect intercellular signaling; KEGG enrichment revealed that the microbiome and metabolic disorders had a profound influence on endothelial dysfunction and PE. These findings were consistent with those of previous studies on the mechanism of endothelial dysfunction in PE (Ahmadian et al., 2020; Aneman et al., 2020; Aplin et al., 2020; Vicen et al., 2020). In addition, it was remarkable that several reported PE-associated pathways, including the “Wnt signaling pathway” (Schlosser et al., 2020), “Toll-like receptor signaling pathway” (He et al., 2018), “mTOR signaling pathway” (He et al., 2019), “p53 signaling pathway” (Gao et al., 2016), and “JAK-STAT signaling pathway” (Zhao and Jiang, 2018) were also depicted.

Based on the microarray data and predicted miRNAs, a portion of the ceRNA network was constructed, and GO and KEGG analyses of network-related molecules were performed. The enrichment results emphasized metabolic disorders in PE, in agreement with recent cohort research on metabolomic alterations in postpartum PE plasma (Hong X. et al., 2020); therefore, more research could be done to facilitate the mechanism of PE and women’s long-term cardiovascular disease. In addition, the importance of the p53 and JAK/STAT signaling pathways was reconfirmed. There is no conflict with the former study that proved that p53 upregulation accelerated G1 arrest and apoptosis of HUVECs from preeclampsia patients (Gao et al., 2016), and a placenta microarray analysis also revealed the overrepresentation of the JAK/STAT pathway in PE (Medina-Bastidas et al., 2020), but little direct evidence explained the dysfunction of HUVECs from PE through the JAK/STAT pathway.

Moreover, the following hub genes: *CACNG8*, *LIPG*, *ACAA2*, and *OSMR*, were shown to be involved in the ceRNA network. *CACNG8*, which was confirmed to encode calcium channels implicated in cardiac contraction, was identified as a calcium flux-related gene that depresses ventricular function (Ortega et al., 2015) and might thus account for long-term CVD in preeclampsia patients. *LIPG* can be secreted by endothelial cells and is involved in lipoprotein metabolism. Cytokines greatly affect *LIPG* expression in endothelial cells and are involved in the inflammatory response in various diseases (Hong C. et al., 2020). *ACAA2* plays an essential role in fatty acid metabolism as a key enzyme of fatty acid oxidation (Zhang et al., 2018). *OSMR* is a member of the interleukin 6 receptor family and mainly signals through the JAK/STAT pathway (Hermanns, 2015). Nevertheless, few studies have directly connected the mechanism of PE with these genes.

Finally, we proposed *hsa_circ_0025992* as a novel potential biomarker for the early prediction of PE. According to the MREs prediction, the most significantly changed circRNA *hsa_circ_0025992* might sponge *hsa-miR-20a-3p*, a reported angiogenic microRNA targeting vascular endothelial growth factor (Platania et al., 2019; Maisto et al., 2020), and could thus be involved in the endothelial dysfunction of PE. The area under the receiver operating characteristic (AUC) curve of *hsa_circ_0025992* was 0.8065 [95% confidence interval (CI) 0.6902–0.9210], *P* < 0.001. Compared with *hsa_circ_0036877* (Hu et al., 2018), *hsa_circ_0025992* had a higher specificity of 93.33% and a ΔCt value above 16.36 with a relatively high PPV of 86.7%, whereas the sensitivity was relatively lower. A higher ΔCt value represents a lower expression level; thus, the *hsa_circ_0025992* level in the blood was negatively correlated with systolic blood pressure and diastolic blood pressure. Taken together, *hsa_circ_0025992* can be regarded as a potential biomarker, and the collaborative prediction of multiple factors may facilitate its predictive power.

The main shortcoming of this study was the limited cohort size due to time constraints and strict patient screening criteria. The validation of *hsa_circ_0025992* as a predictive biomarker in more patients from multiple centers is necessary to explore its clinical value. Second, the relationship between *hsa_circ_0025992* and *hsa-miR-20a-3p* needs further research; thus, we will conduct more experiments in the future to illustrate ceRNA function *in vitro* and *in vivo*.

## Data Availability Statement

The datasets presented in this study can be found in the GEO repository (https://www.ncbi.nlm.nih.gov/geo/) with the accession number GSE165324.

## Ethics Statement

The studies involving human participants were reviewed and approved by the International Peace Maternity and Child Health Hospital Ethics Committee. The patients/participants provided their written informed consent to participate in this study.

## Author Contributions

DC and BH analyzed microarray data and performed the experiments. SW, PZ, XZ, and JL provided the material support. DC and XY wrote the manuscript. WC and XY conceived the study and contributed valuable advice to the manuscript. All authors approved the manuscript.

## Conflict of Interest

The authors declare that the research was conducted in the absence of any commercial or financial relationships that could be construed as a potential conflict of interest.

## Footnotes

1 http://cran.r-project.org/

2 http://genome.ucsc.edu/

3 https://blast.ncbi.nlm.nih.gov/

4 http://www.circbase.org/

5 http://www.miranda.org/
