# Supplementary material for: Identification of mRNA-, circRNA- and lncRNA- Associated ceRNA Networks and Potential Biomarkers for Preeclampsia From Umbilical Vein Endothelial Cells
Source: Front Mol Biosci. 2021 Apr 20;8:652250. doi: 10.3389/fmolb.2021.652250 (PMC8093761; doi:10.3389/fmolb.2021.652250)
Supplement: Supplementary file 1 [file Presentation_1.pdf]

Supplementary Material

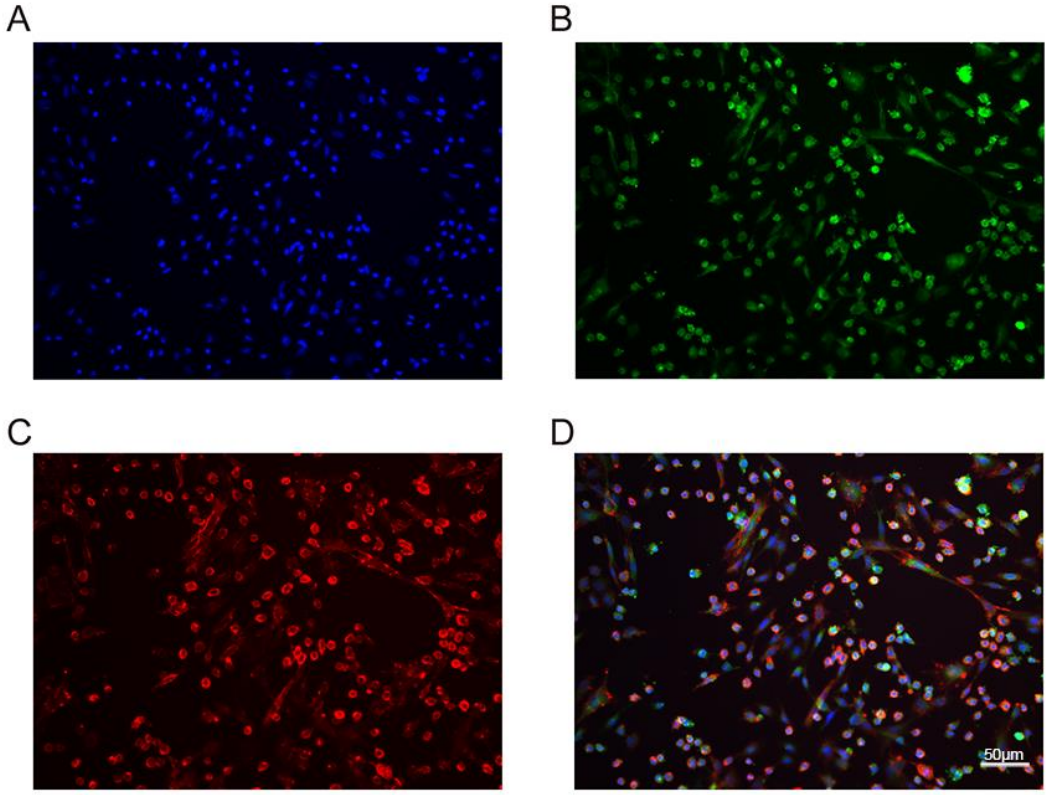

Supplementary Figure 1. Identification of isolated primary HUVECs. The isolated cells were stained with DAPI **(A)**, Factor VIII **(B)** and CD31 **(C).** The merged results showed that the isolated cells were Factor VIII- and CD31-positive **(D)**. Scale bar: 50 μm.

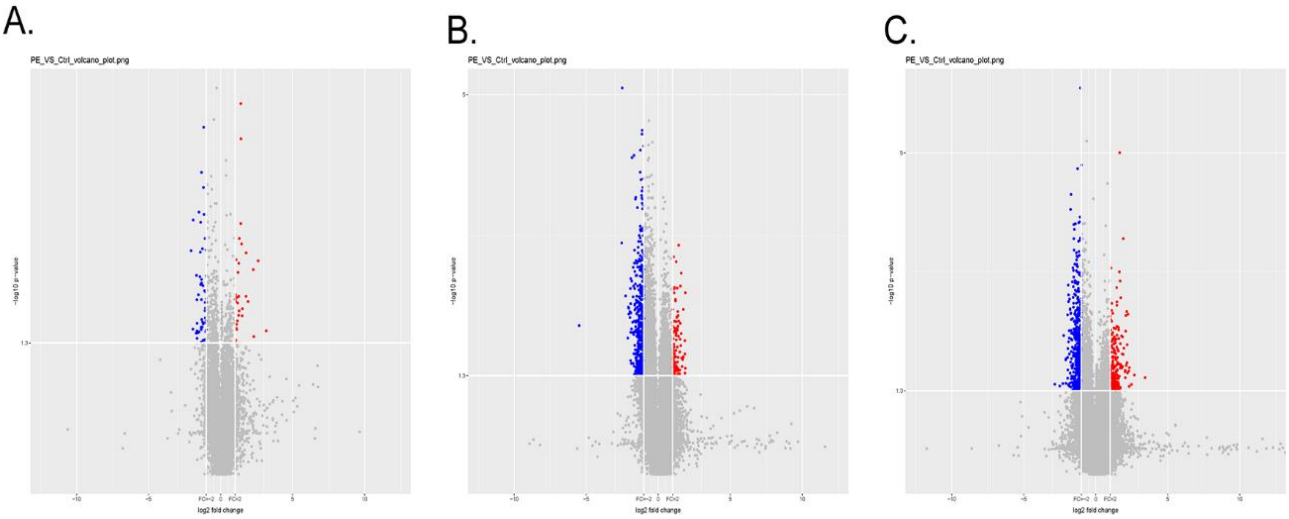

Supplementary Figure 2. The volcano plot of DEmRNAs (A), DEcircRNAs (B), and DElncRNAs (C). In the volcano plot, the log2-fold change is plotted on the x-axis, and the negative log10 P-value is plotted on the y-axis. Blue spots represent downregulated RNAs, and red spots represent upregulated RNAs.

Supplementary Table 1. The predicted MREs of circRNAs

circRNA Name	Regulation	Fold Change	Predicted MREs
hsa_circ_0025992	down	0.327418851	*hsa-miR-20a-3p*
			hsa-miR-4704-5p
			hsa-miR-3667-5p
			hsa-miR-1273g-3p
			hsa-miR-4778-3p
hsa_circ_0090396	down	0.332446308	*hsa-miR-6132*
			hsa-miR-6836-5p
			hsa-miR-3187-5p
			hsa-miR-1285-3p
			hsa-miR-5189-5p
hsa_circ_0066955	down	0.335499799	NA
hsa_circ_0041203	down	0.371189	*hsa-miR-4656*
			hsa-miR-5001-5p
			hsa-miR-4498
			hsa-miR-762
			hsa-miR-4492
hsa_circ_0018116	down	0.378294925	*hsa-miR-4764-3p*
			hsa-miR-6787-3p
			hsa-miR-544a
			hsa-miR-4659a-3p
			hsa-miR-4659b-3p

NA： not available.
